# Pennsylvania Hospital

**Published:** 1838-02-14

**Authors:** 


### CLINICAL LECTURES.

### PENNSYLVANIA HOSPITAL.

*Wednesday, Jan. 24th.*—Dr. Coates remarked that he proposed to-day to call the attention of the class to the consideration of two or three remedies, which had lately acquired some notoriety in the hands of Thompsonian practitioners. Quackery, said he, we can never hope altogether to overturn. Like the poor we seem destined to "have it always with us;" nor do I conceive that the superiority of the regular physician consists in his certificate or diploma, but in a greater degree of knowledge and integrity. I have been induced to make trials of one or two of the articles to which I have alluded, from the fact, that at a recent trial in New York, it appeared that physicians were utterly unfamiliar with them; and I know no reason why powerful medicinal agents should be abandoned by the honourable and skilful to the ignorant and designing. I have therefore searched for cases, fit for the administration of these remedies, and have met with two, in one of which the article used produced some good results, and in the other none.

The *lobelia inflata* is the first article, the consideration of which I shall undertake. It was known in the materia medica, long before an individual named Abraham Thompson obtained notoriety by the exclusive application of it to all diseases. It is one of a genus of plants, the properties of the different species of which are well known to be emetic and narcotic. One of these is the lobelia syphilitica, which is however now little used; and seems never to have been much employed, except for gonorrhœa. It seems to me probable that the genus lobelia has not had full justice done it. Its properties are not sufficiently well known, having been set down as the same as those belonging to tobacco. The order lobeliaceæ resembles very nearly that of the campanulaceæ; and still more that of the compound flowers; among which we do not in general find violent poisons.

[Dr. Coates here exhibited a drawing of the lobelia inflata.]'

He continued: At the trial of Abraham Thompson, it was deposed that he was in the habit of making use of often repeated emetic doses of the lobelia inflata. I may remark that Dr. J. R. Coxe states that he has seen death result from the use of the lobelia, without its producing vomiting. The Thompsonian course consists in a tea-spoonful of the lobelia every fifteen minutes, till it produces vomiting. This is followed by the steam-bath, and afterwards by the administration of what Thompson is stated to have originally called his *coffee;* but which is now styled "composition tea." This, I have been informed, is a strong decoction of a mixture of red pepper, mustard, cloves, and the candleberry myrtle; or as they call it, the bay-berry root bark.

At the recent trial at New York, it was deposed that the *bay-berry root bark* is a poison; but it was alleged that the Thompsonians administer large quantities of it with impunity. The popular designation of this plant, (bay-berry,) is, I think, very incorrect. The term bay is universally applied to the laurel. One of our earliest authorities for this, and there can be no better, is the English version of the scriptures. I would therefore reject this name bay-berry, which is a mere local usage of a part of New England. I exhibit to you, gentlemen, the bark of the stem of this plant: the bark of the root I could not obtain, without applying to sources to which it would be unpleasant to address myself. The Shakers have usually the best assortment of our native medicinal plants, to be met with. The bay-berry is described as being very acrid, even astringent in its taste; these qualities you can now test by personal observation.

I administered, in my recent trials, the powdered lobelia in the dose of about thirty grains, and with very great benefit to the man, Rogers, who is labouring under severe asthma, with dilation of the bronchiæ. The tincture of lobelia has long been administered, as you know, in cases of dry asthma. A patient formerly in the hospital has been relieved by the use of it, for a series of winters. The dose of it has been from ten to forty drops, three times a day; and, sometimes, it may be pushed to larger doses. The Rev. Dr. Cutler, of Massachusetts, first called our attention to this remedy. Dr. Bigelow, of Boston, states that he has used it in table spoonful doses. I cannot well reconcile this discrepancy: and this circumstance strongly points out the necessity of extending our observations. The tincture is made from the fresh plant. When I was apothecary in this house, I remarked that some tinctures of recent vegetables were made much weaker from being diluted with the watery juices of the plant. Dr. W. P. C. Barton states, in his Botany, that the tincture of the fresh lobelia is stronger than that from the dry. This is contrary to the remark which I have just made.

The lobelia in over doses causes dizziness, nausea, and the other usual effects of so many narcotics. Dr. Smith (the resident physician,) and myself watched the effects that were produced in the man Rogers, from immediately after the administration of the remedy. The dose was one tea-spoon-full; and a similar spoonful weighed 29 grains. In four minutes after he had taken it, active vomiting was produced, which, at the end of twelve minutes, was entirely over.* The matter vomited was water tinged green from the colouring matter of the lobelia. No head-ache nor dizziness occurred: the only result was a diminution of the cough, and of the dyspnœa and anxiety. The absence of the bad symptoms is probably owing to the short time which the lobelia remained in the stomach. In order for a narcotic to produce its poisonous effects, it must of course be retained. Orfila, you know, in making his experiments with poisons, invariably tried the œsophagus, to prevent vomiting. Taking this into consideration, we may be struck with a nearer view of the resemblance of these to ipecacuanha, and other more familiar emetics.

* It is since ascertained that he had two more discharges after an interval.

These also possess the power of producing nausea, salivation, dizziness, weakness, coldness, sweat, and other symptoms commonly attributed to poisoning; and if long retained and in considerable quantity, these phenomena may become alarming.

The matter vomited, after an ordinary dose of the lobelia, is a little mucus, some saliva, and water tinged with the medicine. If the dose be large, greenish or blackish looking bile comes up. This matter the Thompsonians call *canker*. They frequently impress upon their patients the belief that it is some poisonous matter, the cause of their disease, and that recovery depends upon the getting rid of it.

The lobelia then is a very powerful remedy,—much too powerful for physicians to neglect it and suffer the employment of it to give vogue to empiricism. Of the effects of such a line of conduct, at least in our city, you have an example in the history of the *sarsaparilla*. It once enjoyed a great reputation: after a while, our physicians in a great measure restricted its use to the form of copious decoctions; some authorities of eminence having ascribed all its beneficial effects to the large quantity of water usually taken with it. In this manner the syrups and extracts went out of vogue; until Mr. Swaim took it up, and turned its undeniable powers into a source of emolument,—greatly promoted from the regular practitioner having abandoned an old and tried remedy.

I believe that the former prevalence of Broussaisism, and his ideas of the great frequency and importance of gastro-enteritis have had much to do with the disuse of articles of this class, when gastric inflammation was considered as the fundamental source of so many diseases, emetics were of course almost universally discouraged. I believe the efficacy of vomiting is pretty generally now admitted with us. In this class of remedies, the lobelia inflata is certainly worthy of attention. Before dismissing the subject, I show you the powder of the lobelia: it is extremely irritating in its properties, readily excites cough, and has even produced salivation of an hour's duration by the inhalation of a little of the dust. Of course much caution is required, in the use of such a remedy.

The Candleberry Myrtle, or myrica cerifera, is a member of not a very numerous class of plants—the myriceæ. No very active plant is closely allied to the one under notice, nor are the botanical characters of the tribe very strongly marked. Perhaps we may sum up the botanical affinities by saying that they throw no light on its medical qualities. It is stated that, in its properties, it is emetic and strongly astringent. That it is acid, is apparent from chewing it. I gave it once here, in a case of diarrhœa, but it disagreed with the patient, producing vomiting and increasing purging; and I stopped the use of it. I am very unfavourably impressed with the character of this plant; and entertain much doubt of the propriety and use of making further trials. I will next mention the Trillium, or Beth-root. This plant is to such a degree unknown, that, at the late trial, one of the Thompsonian practitioners was called upon to spell the name. It is habitually and extensively used by the Shakers, and I believe by some physicians or at least practitioners. The Shakers send us Trillium atropurpureum. The cernuum grows near Philadelphia.

I have not given it to any patient. It is said to be narcotic, anodyne, astringent, restringent and pectoral. Proper cases for its exhibition would be hœmorrhages, or where profuse discharges are to be arrested.

It has been said that the fewer the articles of the Materia Medica, the better. This I grant, for habitual use; but it is surely proper to be acquainted with all. If no remedies used by unlearned practitioners had been tried in modern times, we should be without ipecachuanha and cinchona. It is false delicacy to refrain from using whatever has been touched by quacks. I for one will never be deterred from promulgating truth, by any such scruples.

